# Structural and dynamic studies reveal that the Ala-rich region of ataxin-7 initiates α-helix formation of the polyQ tract but suppresses its aggregation

**DOI:** 10.1038/s41598-019-43926-9

**Published:** 2019-05-16

**Authors:** Jun-Ye Hong, Dong-Dong Wang, Wei Xue, Hong-Wei Yue, Hui Yang, Lei-Lei Jiang, Wen-Ning Wang, Hong-Yu Hu

**Affiliations:** 1 State Key Laboratory of Molecular Biology, Shanghai Institute of Biochemistry and Cell Biology, Center for Excellence in Molecular Cell Science, Chinese Academy of Sciences, Shanghai, 200031 P.R. China; 2 Shanghai Key Laboratory of Molecular Catalysis and Innovative Materials, Department of Chemistry, and Institutes of Biomedical Sciences, Fudan University, Shanghai, 200433 P.R. China; 3 University of Chinese Academy of Sciences, Beijing, 100049 P.R. China

**Keywords:** Protein aggregation, Molecular biophysics

## Abstract

Ataxin-7 (Atx7) is a disease-related protein associated with the pathogenesis of spinocerebellar ataxia 7, while its polyglutamine (polyQ) tract in N-terminus is the causative source of aggregation and proteinopathy. We investigated the structure, dynamics and aggregation properties of the N-terminal 62-residue fragment of Atx7 (Atx7-N) by biochemical and biophysical approaches. The results showed that the normal Atx7-N with a tract of 10 glutamines (10Q) overall adopts a flexible and disordered structure, but it may contain a short or small population of helical structure in solution. PolyQ expansion increases the α-helical propensity of the polyQ tract and consequently enhances its transformation into β-sheet structures during amyloid aggregation. An alanine-rich region (ARR) just ahead of the polyQ tract forms a local and relatively stable α-helix. The ARR α-helix can initiate and stabilize helical formation of the following polyQ tract, but it may suppress aggregation of the polyQ-expanded Atx7-N both *in vitro* and in cell. Thus, the preceding ARR segment in Atx7-N may influence the dynamic structure and aggregation property of the polyQ tract and even determine the threshold of the pathogenic polyQ lengths. This study may gain structural and dynamic insights into amyloid aggregation of Atx7 and help us further understand the Atx7 proteinopathy based on polyQ expansion.

## Introduction

During protein synthesis, the expanded *CAG* repeats are translated into an uninterrupted series of glutamine residues, which is known as a polyglutamine (polyQ) tract. Such expansion of the polyQ tract can cause protein aggregation and is believed to be the causative source of cytotoxicity and neurodegeneration^1–3^. Information about the structure and dynamics of the polyQ proteins is critical for understanding their aggregation mechanisms and might aid in the development of potential polyQ-disease therapies. However, structure determination of the polyQ region has proven to be an extremely difficult problem^4^. The polyQ region is most likely to adopt β-sheet structures in the aggregates (solid state) according to the information obtained previously^5–10^. There were also many biophysical studies about the soluble monomeric form of polyQ fragments of various length, which adopt random-coil conformation^11,12^, α-helical conformation^13,14^ or β-sheet conformation^14^ depending on the samples used.

Although there were lots of studies that provide important insights into potential conformations of the polyQ-tract sequences, most of them were from artificial synthetic polyQ peptides and performed in the absence of a native protein context. Recent studies indicated that two motifs flanking the polyQ tract greatly influence the aggregation and proteopathy of huntingtin (Htt) exon 1^15–17^. The N-terminal flanking segment with 17 residues has been shown to form marginal or partial α-helix structure that is recognized by HSP90 and enhances Htt aggregation^18^, whereas the C-terminal flanking proline-rich region (PRR) tends to attenuate aggregation^13,15,17^.

There are about nine neurodegenerative diseases (NDs) caused by polyQ expansion of the related proteins^19,20^. Among them, spinocerebellar ataxia 7 (SCA7) is caused by an increased number of *CAG* repeats in the coding regions of the protein ataxin-7 (Atx7)^21,22^. The wild-type Atx7 contains about 10 consecutive glutamines in its polyQ region, while in SCA7 patients the polyQ tract extends to more than 36 residues^23^. Human Atx7 is a component of the deubiquitination module (DUBm) in SAGA (Spt-Ada-Gcn5-Acetyltransferase) complex for transcriptional regulation^24^. Our previous studies revealed that sequestration of R85FL/ponsin by the polyQ-expanded Atx7 in cell is mediated by interaction of the third SH3 domain of R85FL with PRR of Atx7^25^, and aggregation of polyQ-expanded Atx7 specifically sequesters ubiquitin-specific protease 22 (USP22) and compromises its deubiquitinating function in SAGA complex^26^. Several studies revealed that proteolytic processing of Atx7 by caspase-7 may contribute to the disease pathogenesis of SCA7^27–29^.

**Figure 1:**
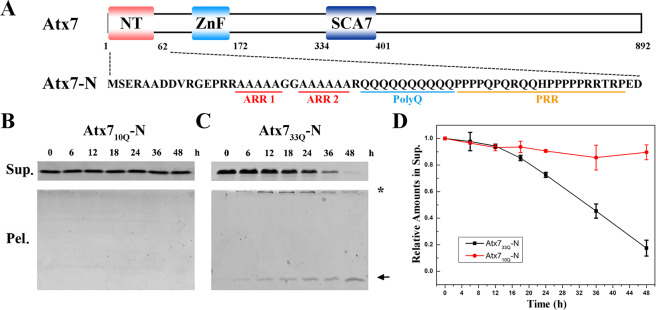
PolyQ expansion enhances aggregation of Atx7-N. (**A**) Domain architecture of Atx7 and its N-terminal sequence. NT, N-terminus; ZnF, zinc finger; SCA7, SCA7 domain. Atx7-N, the N-terminus of Atx7 (residues 1–62); Atx7-N172, the N-terminal 172 residues of Atx7. ARR, alanine-rich region consisting of ARR1 and ARR2 motifs; polyQ, polyglutamine tract; PRR, proline-rich region. (**B**,**C**) Supernatant/pellet fractionation assay for aggregation of Atx7_10Q_-N (**B**) and Atx7_33Q_-N (**C**) during incubation. *Sup*., supernatant; *Pel*., pellet. The major bands for aggregates are indicated with an arrow, while the SDS-resistant aggregates of large molecular weights are marked with a star. The incubation was carried out at 37 °C with continuous shaking and a protein concentration of 100 μM in a PBS buffer (50 mM phosphate, 50 mM NaCl, pH 7.0). (**D**) Quantification of the amounts of Atx7_10Q_-N and Atx7_33Q_-N in supernatant. Data are shown as Means ± SEM (n = 3).

Atx7 is a relatively large protein of 892 amino-acid residues. The N-terminal 62-residue fragment of Atx7 (Atx7-N) contains a polyQ tract, a PRR segment, and an alanine-rich region (ARR) (see Fig. 1A). We investigated the structure, dynamics and aggregation properties of Atx7-N by biochemical and biophysical techniques. We observed that Atx7-N may form marginally stable α-helical structures that are rather dynamic and flexible. PolyQ expansion increases the helical structures and enhances aggregate formation. The ARR segment initiates and stabilizes the helical structure of the polyQ tract, but it suppresses the amyloid aggregation. This study will help us gain mechanistic insights into the polyQ aggregation and further understand the Atx7 proteinopathy based on polyQ expansion.

## Results

### PolyQ expansion enhances aggregation of Atx7-N

Besides polyQ expansion, the N-terminal proteolytic fragments of Atx7 are also considered as a causative pathogen of SCA7 in transgenic mouse model^27^. We prepared the N-terminal 62-residue fragments of Atx7 (Atx7-N) (Fig. 1A) with different polyQ lengths by recombinant techniques and purified these proteins *in vitro*. Then we performed the supernatant/pellet fractionation experiment and quantitated the protein amounts in supernatant during incubation (Fig. 1). While the amount of the 10Q protein (Atx7_10Q_-N) remained unchanged in supernatant (Fig. 1B,D), that of the 33Q protein (Atx7_33Q_-N) decreased gradually with the incubation processing (Fig. 1C,D). Accordingly, the amount of Atx7_33Q_-N in pellet was increased considerably (Fig. 1C). Compared with the normal Atx7_10Q_-N protein that did not form aggregates during incubation, Atx7_33Q_-N readily aggregated into precipitates *in vitro*. This result suggests that polyQ expansion accelerates aggregation of Atx7-N, in consistent with the previous observation on full-length Atx7^30–32^.

### Atx7-N forms marginally stable α-helices and the helical propensity increases with polyQ expansion

**Figure 2:**
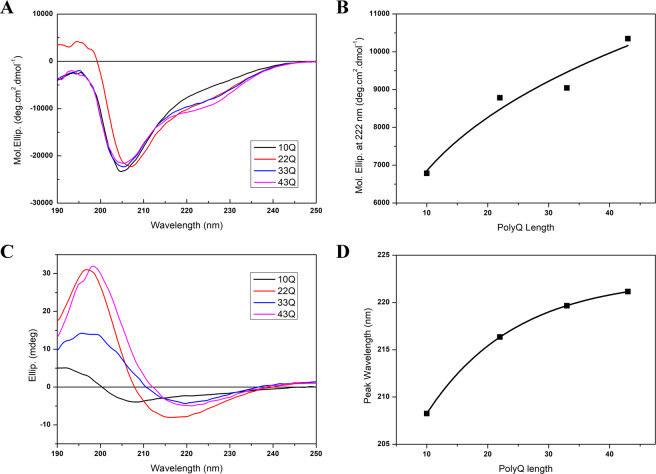
Characterization of the secondary structures of Atx7-N variants by circular dichroism. (**A**) Far-UV CD spectra of the Atx7-N variants with different polyQ lengths in solution. The spectra were acquired at 25 °C and a protein concentration of 0.2 mg/mL in a PBS buffer (50 mM phosphate, 50 mM NaCl, pH 7.0). (**B**) Plot of the ellipticity at 222 nm versus polyQ length. Data are shown as molar ellipticities (deg.cm^2^/dmol). (**C**) Solid-state CD spectra of the Atx7-N variants with different polyQ lengths. The spectra were acquired on a thin protein film at 25 °C. (**D**) Plot of the maximal wavelength of the negative peak versus polyQ length. The negative peak shifts from a wavelength of 202 nm to 220 nm with the polyQ expansion.

To understand the intrinsic mechanism of Atx7-N aggregation with polyQ expansion, we applied circular dichroism (CD) spectroscopy to estimate secondary structure changes with the polyQ expansion. The far-UV CD spectra of Atx7_10Q_-N at different concentrations were almost similar at the wavelengths larger than 200 nm (Supplementary Fig. S1), excluding the possibility that the CD spectra recorded are concentration-dependent artifacts. The CD spectrum of Atx7_10Q_-N exhibited a large negative peak at c.a. 203 nm and a small shoulder at around 222 nm (Fig. 2A), suggesting that the normal polyQ-length Atx7-N is intrinsically disordered but may have a small population of marginally stable α-helical structure. With the increase of polyQ length, the shoulder at 222 nm appeared obviously (Fig. 2A,B), indicating that an enlarged population of α-helices formed in Atx7-N with polyQ expansion.

To obtain more structural information of the aggregated forms of Atx7-N, we performed solid-state CD (ssCD) experiment on these Atx7-N proteins with various polyQ lengths. Our laboratory started CD measurements on thin films in 2001^33^ and proved that this method is quite feasible for detecting secondary structures of protein in amyloids^34^. The spectrum of Atx7_10Q_-N in solid state showed a shape indistinguishable from the one in solution (Fig. 2C). However, the three polyQ-expanded species (22Q, 33Q and 43Q) gave quite different ssCD spectra from their respective ones recorded in solution. They exhibited a broad negative peak at 216 nm or the larger and a strong positive peak at 196 nm (Fig. 2C), indicative of structural transformation from a mixture of random coil and marginally stable α-helix to β-sheet structure. Interestingly, the negative peak shifted to a larger wavelength with the increase of polyQ length (Fig. 2D). It implies that, with the expansion of the polyQ tract, Atx7-N aggregates into amorphous amyloids as a consequence of forming typical β-sheet structure.

### Structural ensemble analysis reveals that ARR forms local α-helices and polyQ expansion increases helical population

**Figure 3:**
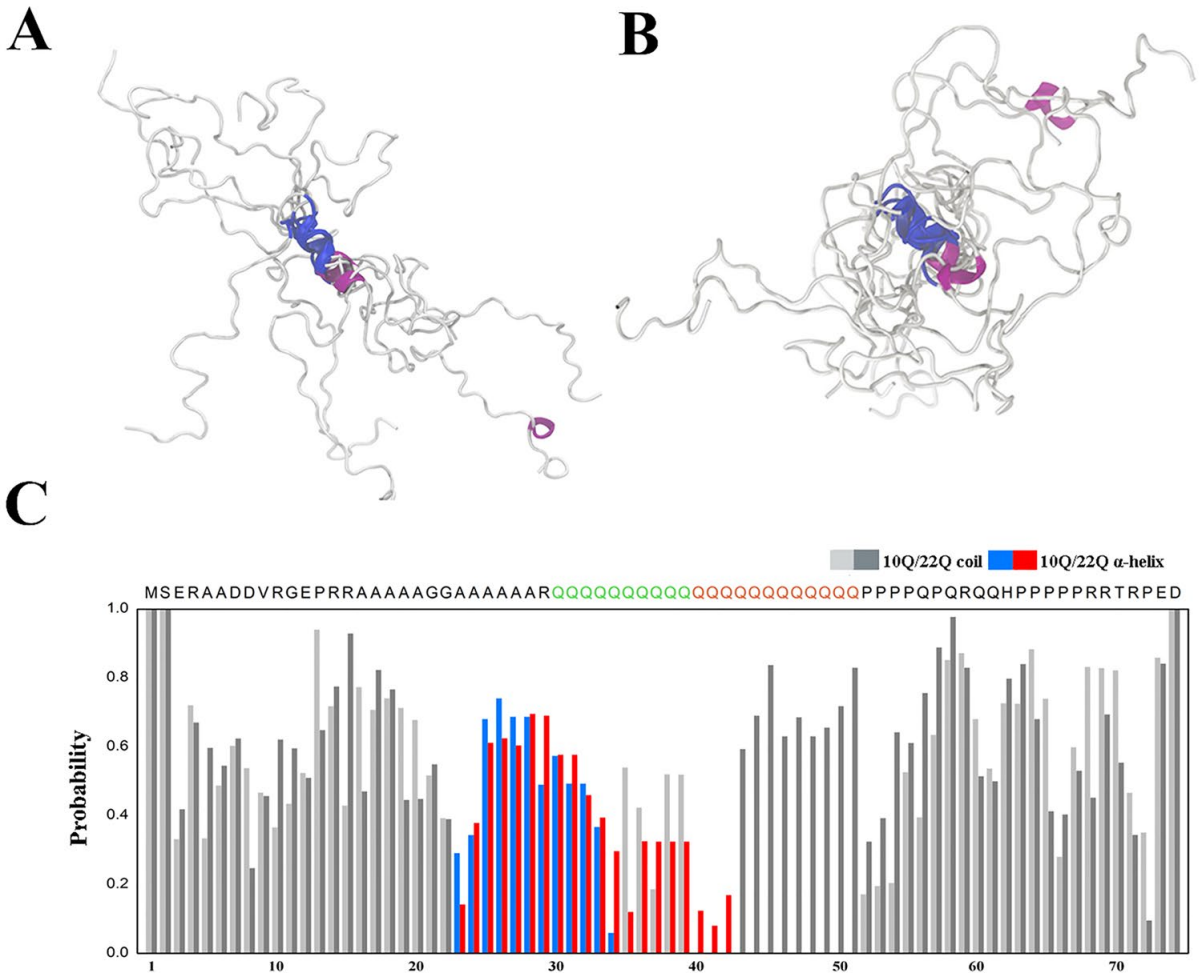
Structural ensemble analysis for Atx7_10Q_-N and Atx7_22Q_-N. (**A**,**B**) The first 6 structures of Atx7_10Q_-N (**A**) and Atx7_22Q_-N (**B**) in their respective ensembles. The helical portion of ARR2 is labeled in blue, and the other helical regions are in pink. (**C**) Secondary structure probabilities derived from structural ensemble analysis highlighting the α-helices of Atx7_10Q_-N (blue) and Atx7_22Q_-N (red) in their polyQ tract regions. The gray and black bars show the random coil probabilities.

To gain more evidence for structural rearrangement and dynamics of Atx7-N in different polyQ lengths, we performed molecular dynamics (MD) simulation^35^ and structural ensemble analysis^36^. The structural ensembles of each Atx7-N system were obtained through these analyses, and their chemical shifts were approximately consistent with those derived from NMR experiment (Supplementary Fig. S2A–D). Figure 3 illustrates the structural ensembles of Atx7_10Q_-N (Fig. 3A) and Atx7_22Q_-N (Fig. 3B). In the structural ensembles, both Atx7_10Q_-N and Atx7_22Q_-N exhibited high propensities of α-helical structure in the ARR region. However, Atx7_22Q_-N had increased α-helical probability in the polyQ region compared with Atx7_10Q_-N (Fig. 3C; Supplementary Fig. S2E,F). With the expansion of polyQ, the α-helices in these regions became longer or higher populated, and the helices formed in both the ARR and polyQ regions combined together to be a long helix. This suggests that the ARR segment forms local α-helices and polyQ expansion increases the helical propensity in Atx7-N.

### The ARR segment forms a local α-helix and initiates helical formation of the polyQ tract

**Figure 4:**
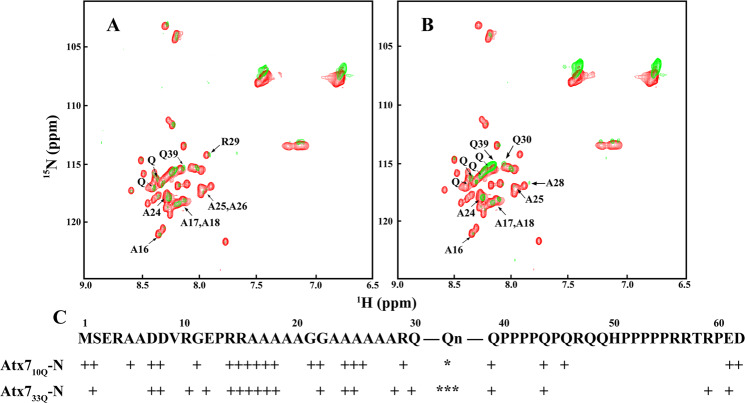
HDX experiment showing that Atx7-N forms partially ordered secondary structures in ARR and polyQ regions. (**A**) Superposition of the HSQC spectra of Atx7_10Q_-N before and after HDX. The spectrum (green) was recorded 15 min after HDX, while the spectrum recorded in H_2_O (red) was set as a control. The assigned remaining peaks are labeled in the spectrum. The protein concentration for HDX experiment was 100 μM in an NMR buffer (20 mM phosphate, 50 mM NaCl, pH 6.5). (**B**) Same as (**A**), Atx7_33Q_-N. Additional peaks for unassigned Gln residues are also indicated. (**C**) Comparison of the exchange rates for the amides of backbone residues. +, the amide of assigned residue with slow HDX. *The amide of unassigned Gln with slow HDX in the polyQ region. The residues are numbered in the sequence with the normal polyQ length (10Q) of Atx7-N.

To obtain detailed information on the structural rearrangement with polyQ expansion, we investigated the Atx7-N species with different polyQ lengths at atomic resolution by NMR techniques. The backbone chemical-shift assignments for Atx7_10Q_-N were achieved by triple resonance experiments and sequential assignments, as shown in the ^1^H-^15^N heteronuclear single quantum coherence (HSQC) spectrum (Supplementary Fig. S3). The backbone chemical shifts of Atx7_33Q_-N except for the polyQ region were derived from overlapping and comparing the spectra with those of Atx7_10Q_-N. We then performed hydrogen/deuterium exchange (HDX) experiment and identified the remaining amide peaks in the HSQC spectra. The HDX data showed that a large number of peaks weakened or disappeared quickly both in Atx7_10Q_-N and Atx7_33Q_-N, suggesting that both species of Atx7-N are considerably flexible and disordered in solution. However, there were still some peaks remained after HDX; most of them were from the residues located in the ARR and polyQ regions (Fig. 4). It indicates that the backbone amides of the residues in these two regions participate in hydrogen bonding, contributing to formation of the marginally stable secondary structures that slow down the HDX rates. Interestingly, in Atx7_33Q_-N, there were some additional peaks remained from unassigned Gln residues after HDX (Fig. 4B). Overall, the HDX rate of Atx7_33Q_-N is slightly smaller than that of Atx7_10Q_-N in the polyQ region and its flanking residues (Fig. 4C). Although we cannot specify the hydrogen bonds formed whether via intra- or inter- molecular interactions or involved in which secondary structure types (α-helix, β-sheet, etc.), the HDX experiment demonstrates that the ARR segment forms a local secondary structure and polyQ expansion enhances structural formation especially in the flanking residues of the polyQ tract.

**Figure 5:**
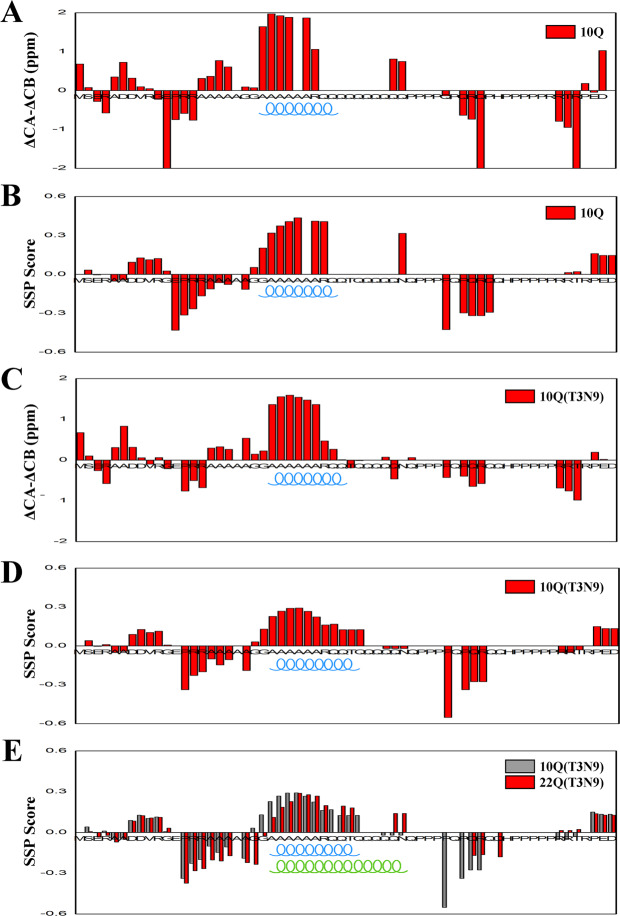
Secondary-structure prediction based on assigned backbone chemical shifts. (**A**) Plot of the (ΔCα-ΔCβ) value versus amino-acid sequence for Atx7_10Q_-N. (**B**) SSP score for Atx7_10Q_-N. (**C**) Plot of (ΔCα-ΔCβ) versus amino-acid sequence for the T3N9 mutant of Atx7_10Q_-N. (**D**) SSP score for the T3N9 mutant of Atx7_10Q_-N. (**E**) Comparison of the SSP scores of Atx7_10Q_-N (T3N9) and Atx7_22Q_-N (T3N9). The helical segments are indicated in the graphs. In SSP program, a score of +1 denotes a well-formed helix while a score of -1 is for the well-formed extended strand.

To obtain more structural features of the polyQ region, we attempted to further assign the chemical shifts in this region by intercalating a T3N9 mutation, in which the third and ninth Gln residues in the polyQ region were substituted by Thr and Asn respectively. We firstly obtained the backbone chemical shifts of the Gln residues around the Thr and Asn mutations by sequential assignments (Supplementary Fig. S4), then we performed secondary structure prediction (Fig. 5) based on the assigned backbone chemical shifts in Atx7_10Q_-N and Atx7_22Q_-N (Supplementary Fig. S5). As known, the absolute value of (ΔCα-ΔCβ) is a sensitive indicator of the residual secondary structures in disordered proteins^37^. The small absolute (ΔCα-ΔCβ) values indicated that Atx7_10Q_-N is overall lacking stable secondary structures (Fig. 5A), in consistent with our CD result (Fig. 2A). Nevertheless, several regions have relatively large deviations in the secondary chemical shifts. For example, residues Ala24 - Ala28 within the ARR2 motif had the (ΔCα-ΔCβ) values larger than 1 ppm (Fig. 5A,C), suggesting that this region is populated with a helical conformation to some degree. To gain quantitative insights into the populations of different helical structures, we further analyzed all the backbone chemical shifts (NH, N, Hα, Cα and Cβ) by SSP (secondary structure prediction) program^38^. All absolute values of the SSP scores were less than 0.5 (Fig. 5B,D), suggesting that the overall Atx7_10Q_-N protein has little stable secondary structure. Nevertheless, the SSP scores in the ARR2 region were larger than 0.3, while those in the Arg29 - Gln33 region were larger than 0.15, implying that these regions are populated with helical conformations, probably marginally stable α-helices. We also compared the SSP scores of the T3N9 mutants with different polyQ lengths. In general, these variants gave similar patterns in SSP score profiles (Fig. 5E), indicating that their secondary structures are quite similar. However, Atx7_22Q_-N had higher SSP scores at Gln37 and Asn38 (>0.15), whereas Atx7_10Q_-N exhibited negative values at these residues (Fig. 5E). It implies that the consecutive Ala residues ahead of the polyQ tract form an α-helical structure, while polyQ expansion elongates the α-helix or prompts the helical population in the following Gln residues.

### The ARR2 helix suppresses aggregation of the polyQ-expanded Atx7-N

**Figure 6:**
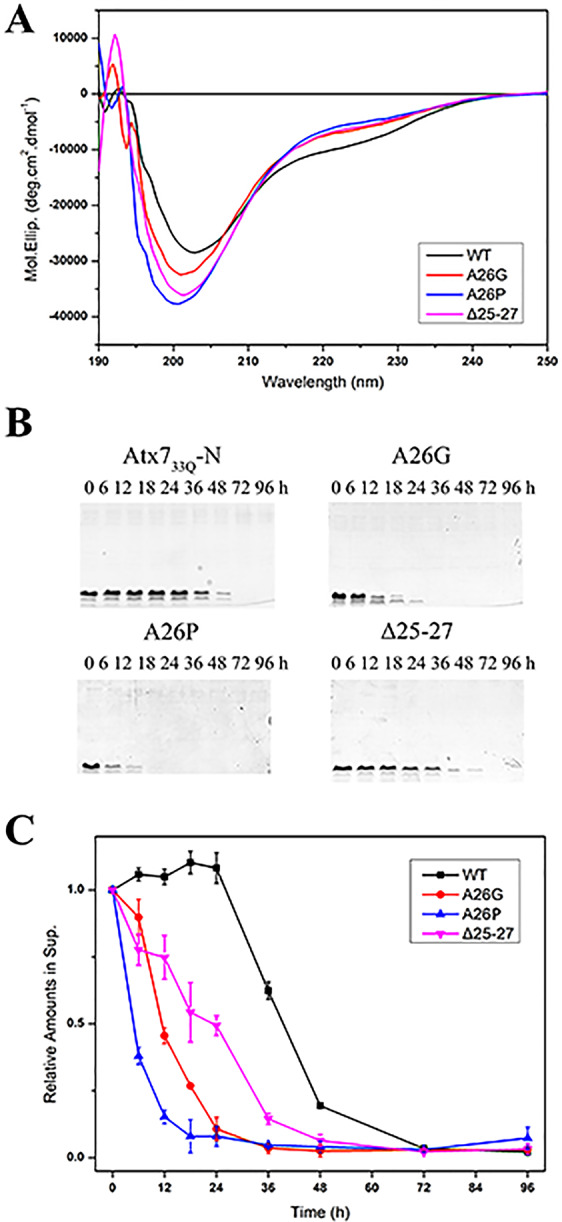
ARR forms relatively stable α-helix structure but suppresses aggregation of the polyQ tract. (**A**) Far-UV CD spectra of Atx7_33Q_-N and its ARR mutants (A26G, A26P and Δ25–27). All the ARR mutants showed the CD spectra with their shoulders disappeared around 222 nm. (**B**) Supernatant/pellet fractionation assay for Atx7_33Q_-N and its helix-disrupting mutants. The protein concentration was 100 μM in a PBS buffer (50 mM phosphate, 50 mM NaCl, pH 7.0). (**C**) Time courses showing aggregation of Atx7_33Q_-N and its helix-disrupting mutants. The aggregation abilities were represented by the relative amounts of Atx7_33_-N species in supernatant. Data are shown as Means ± SEM (n = 3).

The ARR segment can form relatively stable α-helical structure and thus initiate helical formation of the polyQ tract. To evaluate the impact of the ARR helix on Atx7-N aggregation, we prepared three mutations (A26G, A26P, Δ25–27) in Atx7_33Q_-N, a pathogenic species, in order to disrupt the α-helix structure in the ARR2 region. These mutants were expressed with a thioredoxin (Trx) fusion and purified after removal of the Trx tag. The CD spectra showed that, compared with the wild type, all these Atx7_33Q_-N mutants had their shoulders disappeared at around 222 nm (Fig. 6A), suggesting that the α-helices formed by ARR2 had been disrupted by mutation. We then measured the aggregation processes by supernatant/pellet fractionation immediately after cleavage of the soluble Trx tag. Our result showed that all the three Atx7_33Q_-N mutants with their ARR2 helix disrupted aggregated into precipitates much faster than the wild type (Fig. 6B,C). It seemed that A26P reached its saturation of aggregates rapidly, then A26G. The aggregation of Δ25–27 was not rapid as expected, but it still had a strong propensity of aggregation. Taken together, these data demonstrate that the ARR2 motif retards aggregation of polyQ-expanded Atx7-N through formation of marginally stable α-helical structure.

**Figure 7:**
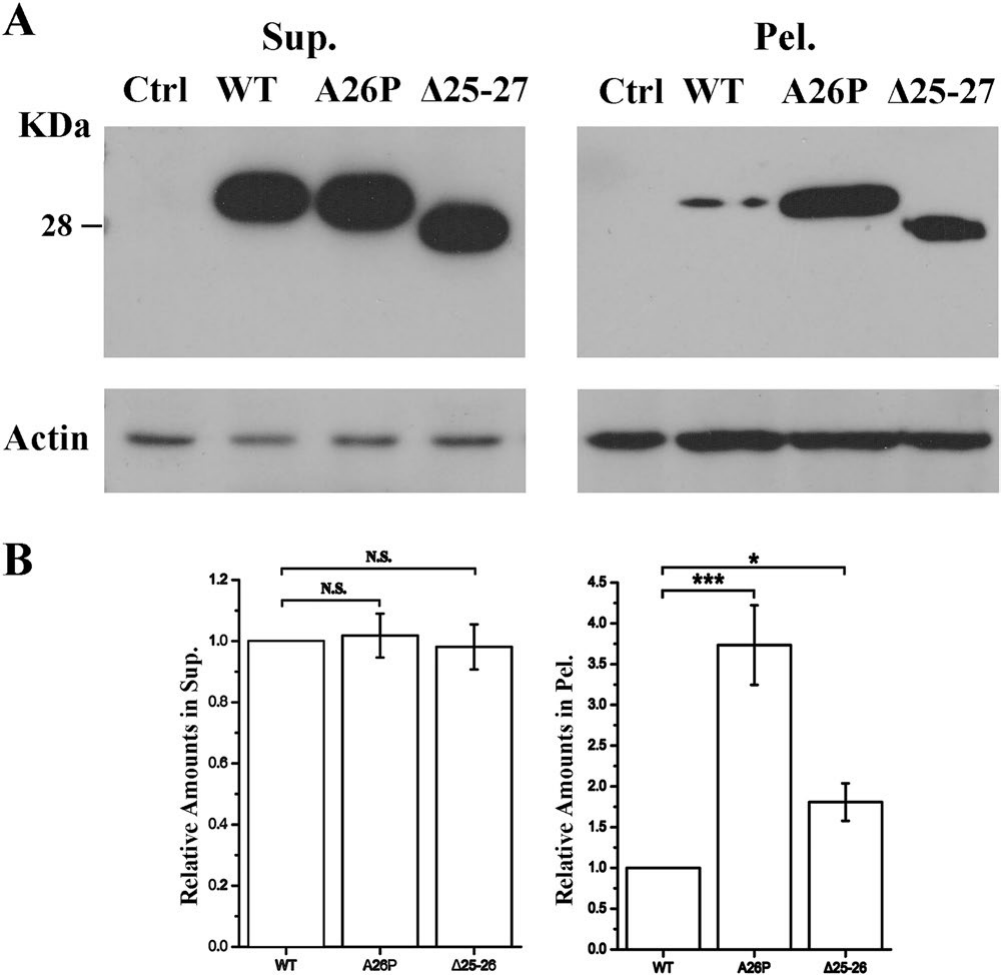
The ARR helix suppresses aggregation of polyQ-expanded Atx7-N172 in cell. (**A**) Supernatant/pellet fractionation assay for Atx7_33Q_-N172 (residues 1–172) and its helix-disrupting mutants. The HEK 293T cells were transfected with FLAG-tagged Atx7_33Q_-N172 or its mutants (A26P and Δ25–26). About 24 h after transfection, the cell lysates were prepared for Western blotting with an anti-FLAG antibody. Ctrl, HEK 293T cell transfected with an empty vector. (**B**) Quantification of the protein amounts in supernatant and pellet respectively. Data are shown as Means ± SEM (n = 3). *p < 0.05; ***p < 0.001; N.S., no significance.

To corroborate the biophysical results under cellular environments, we over-expressed an Atx7_33Q_-N species in HEK 293T cells and performed a supernatant/pellet fractionation experiment (Fig. 7). Because we failed to detect the expression of the N-terminal 62-residue fragments in cell (possibly due to their short sequence or proteolytic degradation), we prepared the expression plasmids harboring the N-terminal 172 residues of Atx7 (Atx7_33Q_-N172)^26^. Compared with wild-type Atx7_33Q_-N172, both mutants (A26P and Δ25–27) especially A26P had large amounts of precipitates in pellet (Fig. 7A,B). As *in vitro*, disruption of the α-helical structure in ARR2 also accelerated the aggregation of Atx7_33Q_-N172 in cell. Thus, we propose that the ARR segment (especially the ARR2 motif) suppresses the Atx7_33Q_-N aggregation through initiating and stabilizing the α-helices in the following polyQ tract and retarding its transformation into β-sheet structure.

## Discussion

The disease-related polyQ proteins are relatively large in size and often composed of multiple domains. Some polyQ proteins, such as Atx7 and Htt, harbor a polyQ tract in their N-termini. They usually undergo proteolytic processing to various fragments catalyzed by proteases (i.e. caspase-7) in cell. The N-terminal fragments containing a polyQ tract are even more cytotoxic or pathogenic^27,28^. For example, Htt exon 1 has been studied extensively and found to be more prone to aggregation and more pathogenic both *in vitro*^39,40^ and in mouse model^41,42^. Besides the polyQ tract^26^ and the PRR motif ^25^, Atx7-N has a special ARR segment (especially ARR2) that tends to form relatively ordered α-helical structure, while the N-terminus of Htt harbors an amphipathic α-helix just ahead of the polyQ tract^43^.

There are several literatures focusing on the study of the polyQ tract and its adjacent sequences. The N-terminal N17 motif of Htt is largely unstructured at neutral pH but adopts an α-helical structure at low pH, which propagates into the polyQ region^44^. The folding equilibrium of N17 is dependent on the polyQ tract, and the more rigid α-helical structure with an expanded polyQ tract may modulate the misfolding process^45^. These observations support the view that the N17 motif of Htt accelerates its aggregation^16,17^. In addition, the N-terminal α-helix in Htt can be recognized by HSP90, which may be beneficial to Htt aggregation in cell^18^. On the contrary, in androgen receptor, the Leu-rich region preceding the polyQ tract causes the polyQ region to form an α-helical structure but protects the protein against aggregation^46^, as in the case of the observation in this study.

We found that the ARR segment of Atx7-N itself forms a relatively stable α-helix and triggers elongation of the helical structure in the following polyQ-expanded tract, and this rigid structure even suppresses the amyloid aggregation of the polyQ-expanded Atx7-N both *in vitro* and in cell. In this aspect, the helical formation of the ARR segment could be regarded as a protective role against Atx7 aggregation. Thus, we deduce that any factors that enhance the helical probability of ARR might be beneficial to suppressing the amyloid aggregation of Atx7.

As known, expansion of the polyQ tract enhances amyloid aggregation of the host proteins^47^. Atx7 is such a polyQ protein, in which expansion of the polyQ tract induces aggregation of the protein and thus causes the pathogenesis of SCA7. We investigated the dynamic structure and structural transformation of the N-terminus of Atx7, and found that, as usual, expansion of the polyQ tract leads to the increase of the secondary structure. Actually, raising the structural probability of a segment will somehow promote structural transformation into β-sheet structures and thus amyloid aggregation into precipitates. On the other hand, multiple consecutive Ala residues will increase the probability of forming α-helical structure in ARR segment. The relatively stable α-helix formed by the local ARR segment may significantly influence the dynamic structure and aggregation propensity of the neighboring polyQ tract region as well as full-length Atx7, and even determine the threshold of the pathogenic polyQ lengths of Atx7.

The multiple Gln segment has a propensity to forming alternatively α-helix and β-sheet structures depending on its context sequence and environment. The marginally stable α-helical structure is beneficial to structural transformation to β-sheet and amyloid aggregation, as in the case of the amyloidogenic core of TDP-43^25,48^. As for the ARR segment, it can form α-helix itself and also initiate α-helical formation of the following polyQ-expanded tract. This may increase the probabilities of forming α-helical structures in the polyQ region and its neighboring sequences. On the other hand, the relatively stable α-helix formed by the ARR segment will probably impede structural transformation itself and even suppresses amyloid aggregation of the polyQ-expanded Atx7-N fragment. So, we propose that the elongated marginally stable α-helical structure in the polyQ tract is the major source for α-to-β structural transformation during amyloid aggregation, whereas the relatively stable α-helix formed by the ARR segment is somehow disadvantageous to structural alteration and amyloid aggregation.

Like the N-terminal fragments of Htt that are more pathogenic than the full-length one^41,42,49,50^, the proteolytic fragments of Atx7 are more amyloidogenic and also contribute to the pathogenesis of SCA7^27^. Generally, the N-terminal segment of Atx7 is flexible and dynamic, but it may contain a considerable population of marginally stable α-helices. Our major finding, in this work, is that ARR of Atx7 initiates α-helix formation of the polyQ tract but suppresses its amyloid aggregation. It will aid in further understanding the mechanisms underlying structural transformation, protein aggregation and cytotoxicity or neurodegeneration, and in developing potential polyQ-disease therapies.

## Materials and Methods

### Plasmids, antibodies and reagents

All constructs used in this study are listed in Supplementary Table S1. The sequences for encoding wild-type Atx7-N (residues 1–62, 10Q) and its polyQ-expanded (22Q, 33Q and 43Q), T3N9 (10Q and 22Q) and ARR (in Atx7_33Q_-N, A26G, A26P and Δ25–27) mutants were PCR-amplified and cloned into a pET-32M vector for prokaryotic expression. For eukaryotic expression, Atx7_33Q_-N172 (residues 1–172) and its ARR mutants (A26P and Δ25–27) were cloned into the FLAG-pcDNA3.0 vector. All the constructs were verified by DNA sequencing. For Western blotting, the antibody against FLAG was from Sigma, and the anti-actin antibody was from Santa Cruz Biotechnology. All secondary antibodies were purchased from Jackson ImmunoResearch Laboratories. PVDF membranes were from PerkinElmer Life Sciences, and the protein bands were visualized using an ECL detection kit (Thermo Scientific).

### Protein expression and purification

All proteins were purified as described previously^18,51^. Briefly, Atx7-N and its variants were purified through a Ni^2+^-NTA column and suffered thrombin cleavage overnight (~8 hrs), and further purified through another Ni^2+^-NTA column to remove the Trx tag. The protein concentration was determined by using the standard BCA assay.

### Time course of aggregation *in vitro*

Before incubation, the protein was centrifuged at 16,200 × *g* for 15 min at 4 °C and sterilely filtered through 0.22-μm filters to remove any granular matter, then was immediately subjected to time-course incubation for examining the aggregation properties. Atx7_10Q_-N and its variants were diluted to 100 μM with a phosphate buffered saline (PBS) (50 mM phosphate, 50 mM NaCl, pH 7.0), followed by incubation at 37 °C with shaking. Time course of the aggregation process was monitored by supernatant/pellet fractionation and SDS-PAGE assay.

### Circular dichroism measurements

The CD spectra were recorded on a Jasco J-715 spectropolarimeter at room temperature. The parameters for recording far-UV CD spectra were as described previously^18^. Each spectrum was obtained through averaging three scans of the same sample. Data were further processed for noise reduction, base-line subtraction, and signal averaging when needed. For solution study, the protein was diluted to a concentration of 0.2 mg/mL (~20 μM) in a PBS buffer (50 mM phosphate, 50 mM NaCl, pH 7.0), and the data were presented as mean residual molar ellipticities (deg.cm^2^/dmol). For solid-state CD measurement^33,34^, ~150 μL of the protein solution (~0.50 mg/mL) was cast onto a 2-cm diameter cylindrical quartz glass for evaporation overnight at room temperature, and then the protein thin film formed on the glass surface was used for ssCD recording. The ssCD spectra were presented as ellipticities (mdeg).

### NMR spectroscopy and chemical shift assignment

All data acquisition was carried out on a Bruker Avance 600-MHz spectrometer equipped with a TCI cryoprobe (Bruker Biospin) at 25 °C. ^15^N/^13^C-labeled protein samples (~400 μM) in a phosphate buffer (20 mM sodium phosphate, 50 mM NaCl, 0.05% w/v sodium azide, pH 6.5, and 95% H_2_O/5% D_2_O) were used for data acquisition. To make sure that the samples were not aggregated during spectra acquisition, newly prepared sample was used for each triple-resonance experiment. To assign backbone chemical shifts, several heteronuclear 3-dimensional spectra, including HNCACB, CBCA(CO)NH, CC(CO)NH, HNHA and ^15^N-NOESY-HSQC, were acquired. The spectra were processed by using NMRPipe and analyzed with Sparky. Thus, most of the non-proline residues in Atx7_10Q_-N were successfully assigned. However, some residues, like Ala19 and the Gln residues in the polyQ region, were hard to be distinguished due to the repeated residues. So we resorted to a mutant T3N9 (the third and ninth Gln residues in the polyQ region were replaced with Thr and Asn, respectively) for further chemical shift assignments. Similar NMR spectra were obtained for the T3N9 mutants of Atx7_10Q_-N and Atx7_22Q_-N. With the help of the T3N9 mutant, we successfully identified some indistinguishable residues, such as Q30, Q33, Q37 and Q39. Based on the chemical shift assignments, the secondary structure prediction was carried out with the (ΔCα-ΔCβ) values^37^ or by the SSP program^38^ according to the literatures.

### Hydrogen/deuterium exchange

As for HDX experiment^52^, the Atx7-N proteins were dissolved in an NMR buffer (20 mM phosphate, 50 mM NaCl, pH 6.5) and their concentration was 100 μM. The protein samples were freeze-dried and re-dissolved with deuterated water (D_2_O), and after duration of 15, 60 or 120 min for H/D exchange at 25 °C, the ^15^N-^1^H HSQC spectra were acquired with the same parameters. The acquiring time for each HSQC spectrum was ~40 min (ns = 16). As controls, the HSQC spectra of the protein samples in the same buffer (in H_2_O) were also acquired. Finally, the slow HDX amides of the residues were identified based on the chemical shift assignments.

### MD simulation and structural ensemble analysis

Through conventional MD and replica exchange MD (REMD) simulations, a large number of structures were firstly generated as a pool for both Atx7_10Q_-N and Atx7_22Q_-N systems. Then the sequential least squares programming (SLSQP) algorithm was applied to assign weights to each structure in the pool to obtain the final structural ensemble. For REMD, we used 8 initial structures, which were previously generated from the structural prediction program I-TASSER^53^, the online server QUARK^54^, or structure modeling with TraDES-2^55^. All simulations were performed using the GROMACS-4.6.5 software package^56^ with the AMBER FF03 force field^57^. Implicit solvent was applied by the Onufriev-Bashford-Case method to calculate the Born radii^58^. The temperatures were maintained using the V-rescale method with a relaxation time of 0.1 ps^59^. All bonds were constrained by the LINCS algorithm^60^. Eight different temperatures ranging from 290–380 K were generated from the Webserver Temperature generator for REMD simulations^61^. The exchange time between two adjacent replicas was 2 ps and each replica lasted for 400 ns. Finally, a total of 3.2 μs simulation time was obtained for each system. The average acceptance ratios of Atx7_10Q_-N and Atx7_22Q_-N were 23.7% and 20.2%, respectively. The last 260 ns of trajectories at 301.89 K were used to construct ensemble. However, this ensemble did not match well with the experimental result, probably due to the force field that caused this mismatch. So we did another 4 conventional MD simulations using AMBER ff99SB force field^62,63^ and 500-ns duration for each simulation. Different approaches have been proposed for deriving representative ensembles such as ENSEMBLE that assigns weights to the different conformations of the pool^35,36^, and ASTEROIDS that relies on a genetic algorithm to select sub-ensembles^37,38^. Herein, the snapshots from REMD and MD simulations were collected and the structures were generated from software TraDES-2^55^ as a pool. Then we used SLSQP algorithm to assign weights to the different conformations of the pool in order to obtain the final structural ensemble. All secondary structure analysis was performed using the DSSP (definition of secondary structure of proteins) program^64^. Chemical shift prediction was performed using SHIFTX2 software^65^. Secondary chemical shift (∆δ), such as ∆δ_Cα_, is defined as: ∆δ_Cα_ = δCα^exp/simu^ − δCα^random^.

### Cell culture and supernatant/pellet fractionation

Human HEK 293T cells were cultured in Dulbecco minimum essential medium (DMEM, HyClone) supplemented with 10% fetal bovine serum (Gibco) and penicillin-streptomycin at 37 °C under a humidified atmosphere of 5% CO_2_. All transfections were carried out by using PolyJet^TM^ reagent (SignaGen). About 24 h after transfection, the cells were lysed in 100 μL of RIPA (radioimmune precipitation assay) buffer (50 mM Tris-HCl, 150 mM NaCl, 1 mM EDTA, 1% Nonidet P-40 and protease inhibitor mixture; pH 7.5) on ice for 30 min and centrifuged at 16,200 × *g* for 15 min at 4 °C. Then the supernatant was added to 100 μL of 2× loading buffer (2% SDS), and the pellet was washed sufficiently with the RIPA buffer three times at 4 °C and added to 40 μL of 4× loading buffer (4% SDS). Finally the samples were subjected to Western blotting analysis.

### BMRB Deposit

The chemical shifts were deposited at Biological Magnetic Resonance Data Bank (BMRB) for Atx7_10Q_-N (No. 27333), Atx7_10Q_-N (T3N9) (27334) and Atx7_22Q_-N (T3N9) (27335).

## Supplementary information

Supplementary Information
