# Author Correction: Structural and dynamic studies reveal that the Ala-rich region of ataxin-7 initiates α-helix formation of the polyQ tract but suppresses its aggregation

**DOI:** 10.1038/s41598-020-66680-9

**Published:** 2020-06-09

**Authors:** Jun-Ye Hong, Wei Xue, Hong-Wei Yue, Hui Yang, Lei-Lei Jiang, Hong-Yu Hu, Dong-Dong Wang, Wen-Ning Wang

**Affiliations:** 1 State Key Laboratory of Molecular Biology, Shanghai Institute of Biochemistry and Cell Biology, Center for Excellence in Molecular Cell Science, Chinese Academy of Sciences, Shanghai, 200031 P.R. China; 2 Shanghai Key Laboratory of Molecular Catalysis and Innovative Materials, Department of Chemistry, and Institutes of Biomedical Sciences, Fudan University, Shanghai, 200433 P.R. China; 3 University of Chinese Academy of Sciences, Beijing, 100049 P.R. China

Correction to: *Scientific Reports* 10.1038/s41598-019-43926-9, published online 16 May 2019

The original version of this Article omitted an affiliation for Jun-Ye Hong, Wei Xue and Hong-Wei Yue.

The correct affiliations for Jun-Ye Hong, Wei Xue and Hong-Wei Yue are listed below:

State Key Laboratory of Molecular Biology, Shanghai Institute of Biochemistry and Cell Biology, Center for Excellence in Molecular Cell Science, Chinese Academy of Sciences, Shanghai 200031, P. R. China

University of Chinese Academy of Sciences, Beijing 100049, P. R. China

This has now been corrected in the HTML and PDF versions of this Article

