# Supplementary material for: Structural and dynamic studies reveal that the Ala-rich region of ataxin-7 initiates α-helix formation of the polyQ tract but suppresses its aggregation
Source: Sci Rep. 2019 May 16;9:7481. doi: 10.1038/s41598-019-43926-9 (PMC6522498; doi:10.1038/s41598-019-43926-9)
Supplement: Supplementary file 1 — Supplementary Information [file 41598_2019_43926_MOESM1_ESM.pdf]

Structural and dynamic studies reveal that the Ala-rich region of ataxin-7 initiates α-helix formation of the polyQ tract but suppresses its aggregation

Jun-Ye Hong, Dong-Dong Wang, Wei Xue, Hong-Wei Yue, Hui Yang, Lei-Lei Jiang, Wen-Ning Wang, Hong-Yu Hu

Suppl. Fig. S1

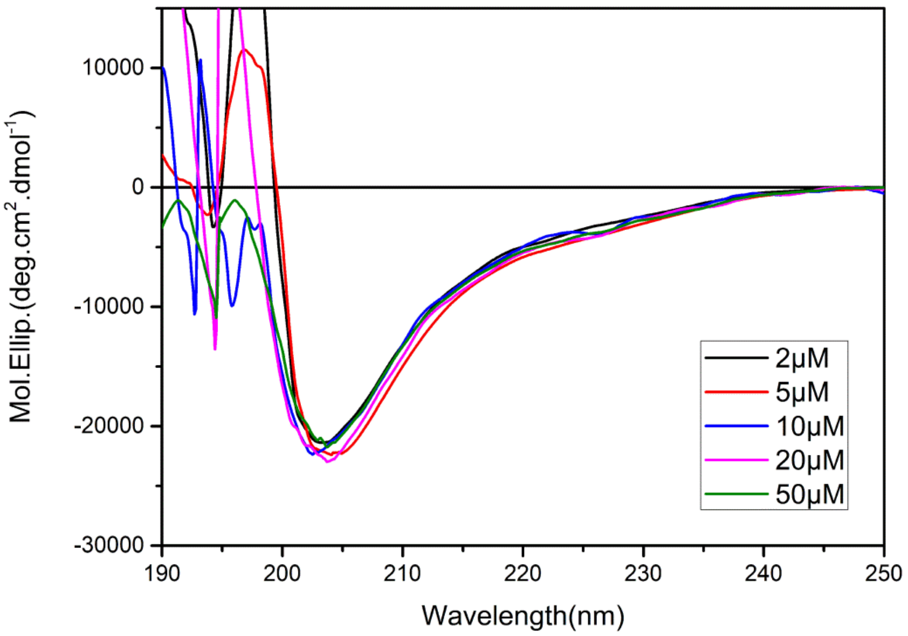

Figure S1. Far-UV CD spectra of Atx7$_{10Q}$-N at different concentrations. The experiments were carried out by using two cuvettes separately with different path-lengths, 1 mm for 10, 20 or 50 μM sample and 10 mm for 2 or 5 μM.

Suppl. Fig. S2

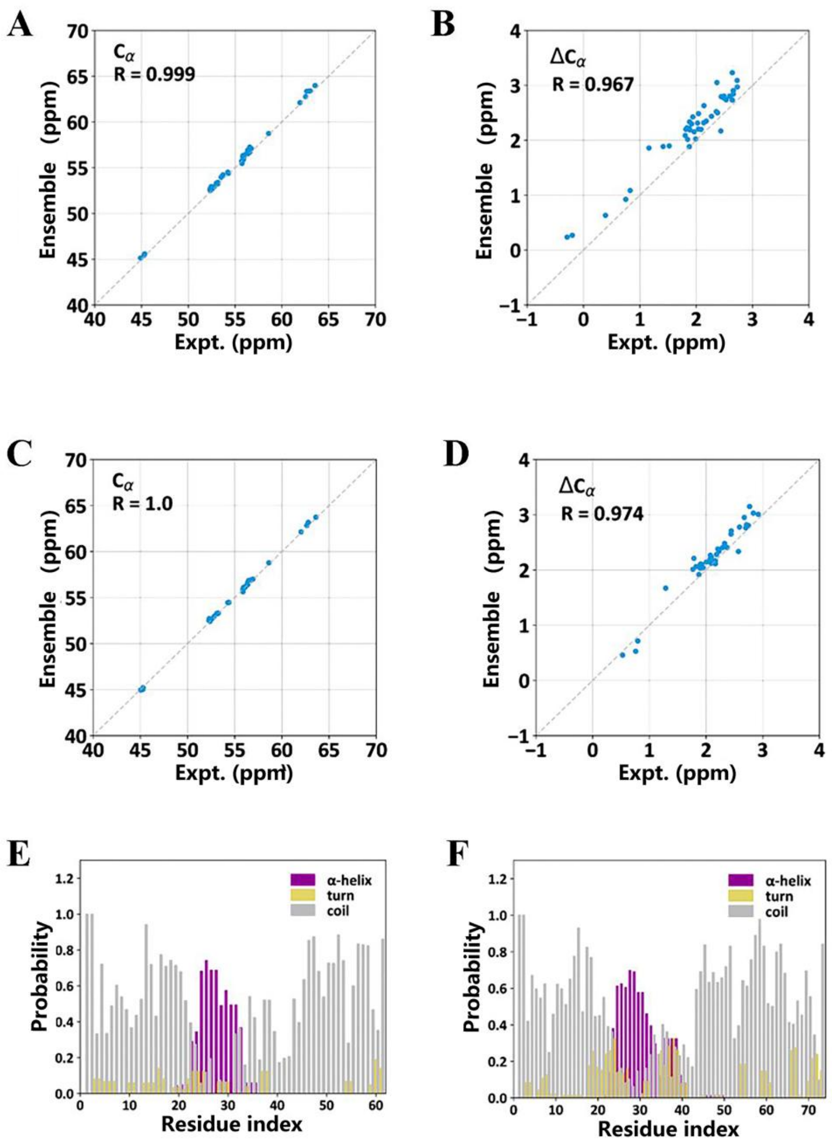

Figure S2. Correlations between the ensemble calculated and experimental chemical shifts. (**A**, **B**) Cα (**A**) and ΔCα (**B**) of Atx7$_{10Q}$-N. (**C**, **D**) Cα (**C**) and ΔCα (**D**) of Atx7$_{22Q}$-N. (**E**, **F**) Secondary structure probabilities of each residue in a sequence alignment for Atx7$_{10Q}$-N (**E**) and Atx7$_{22Q}$-N (**F**).

Suppl. Fig. S3

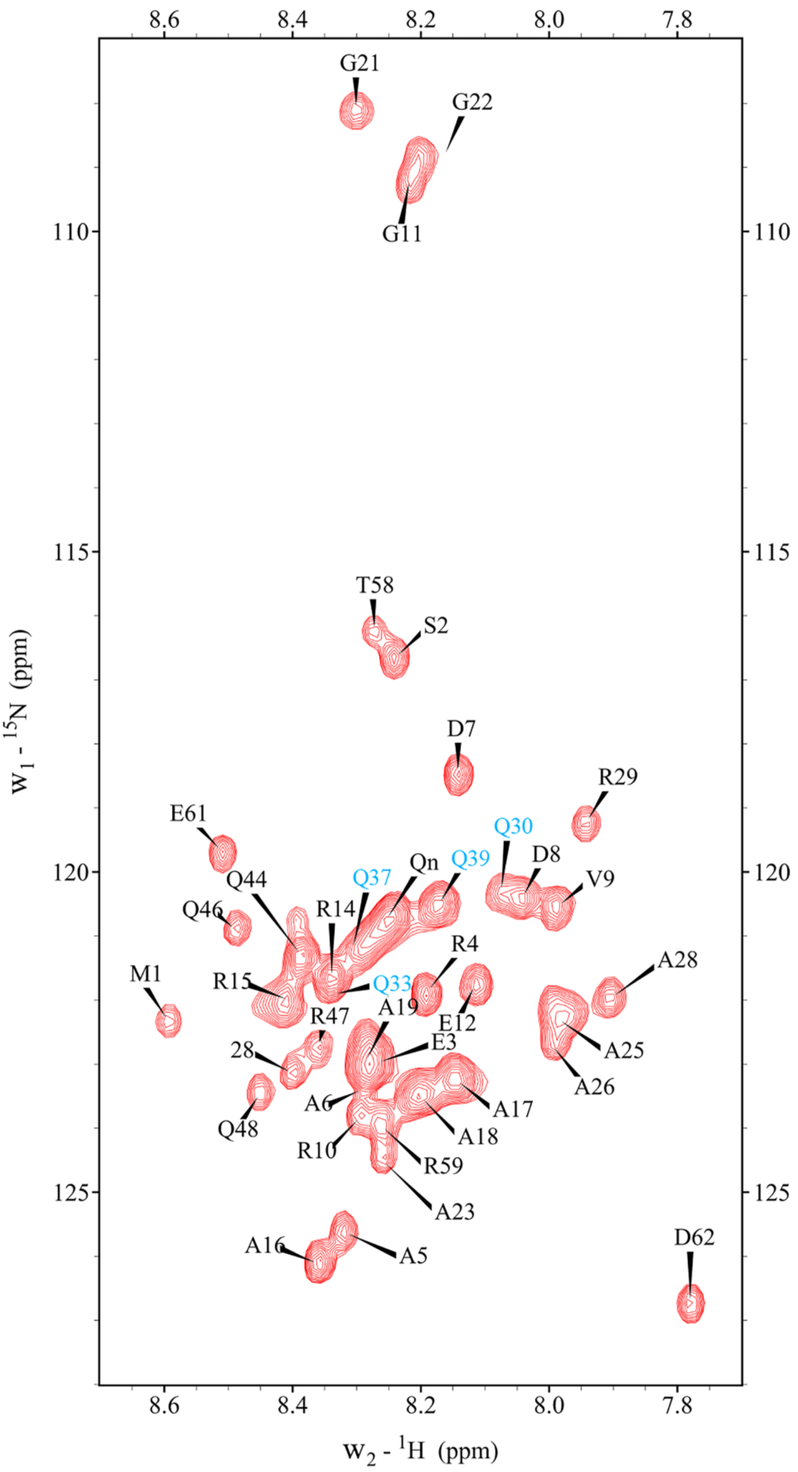

Figure S3. The ^{1}H-^{15}N HSQC spectrum of Atx7$_{10Q}$-N showing the chemical shift assignment. The Gln residues that are assigned through the T3N9 mutant are labeled in blue (See below).

Suppl. Fig. S4

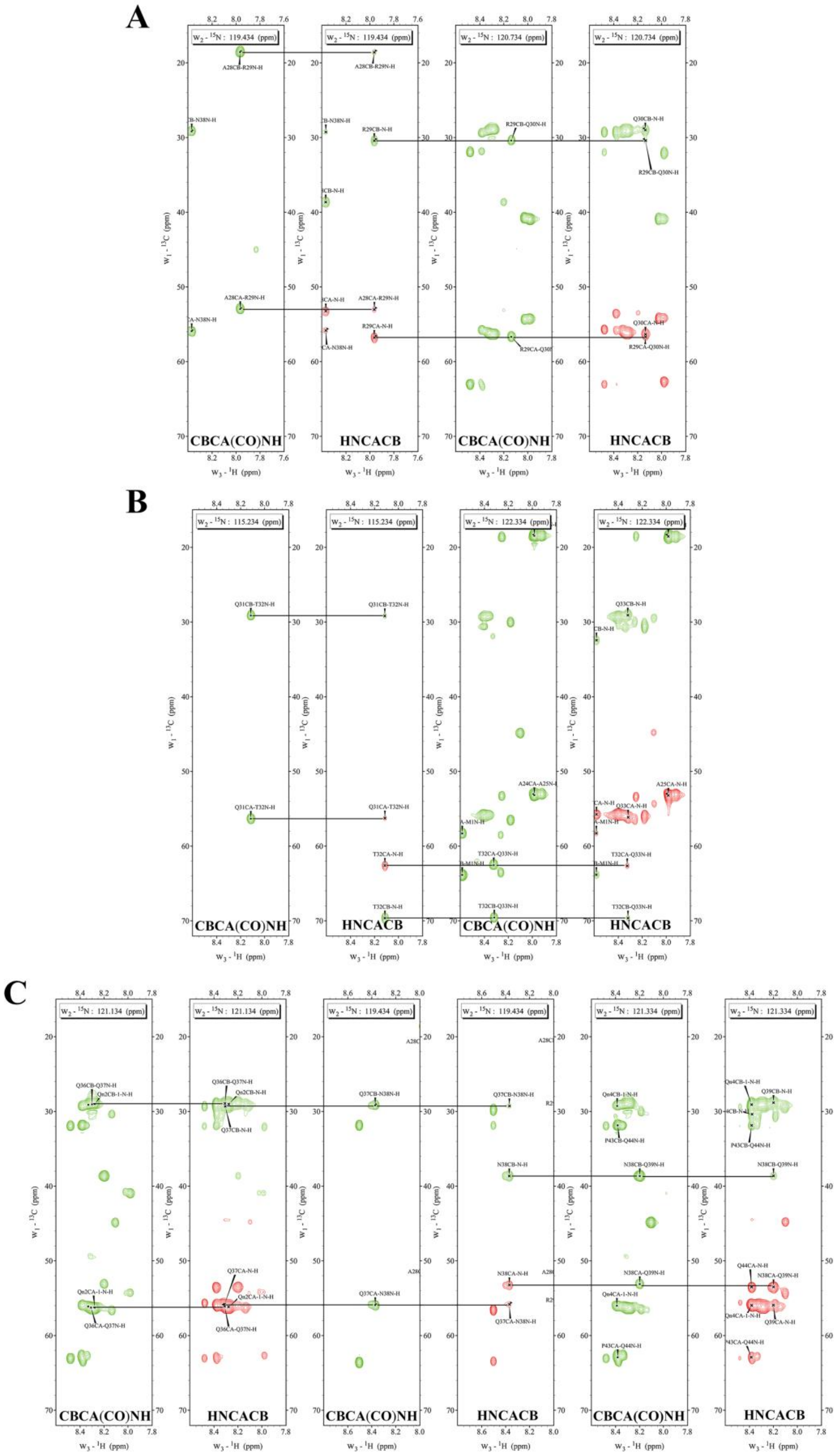

Figure S4. Representative strip plots of the CBCA(CO)NH/HNCACB spectra for sequential assignment of the backbone chemical shifts of the polyQ region in the T3N9 mutant. (**A**) A28R29Q30; (**B**) Q31T32Q33; (**C**) Q36Q37N38Q39. The backbone chemical-shift assignments of the Atx7-N variants were achieved by performing the standard triple-resonance experiments, including CBCA(CO)NH, HNCACB and CC(CO)NH.

Suppl. Fig. S5

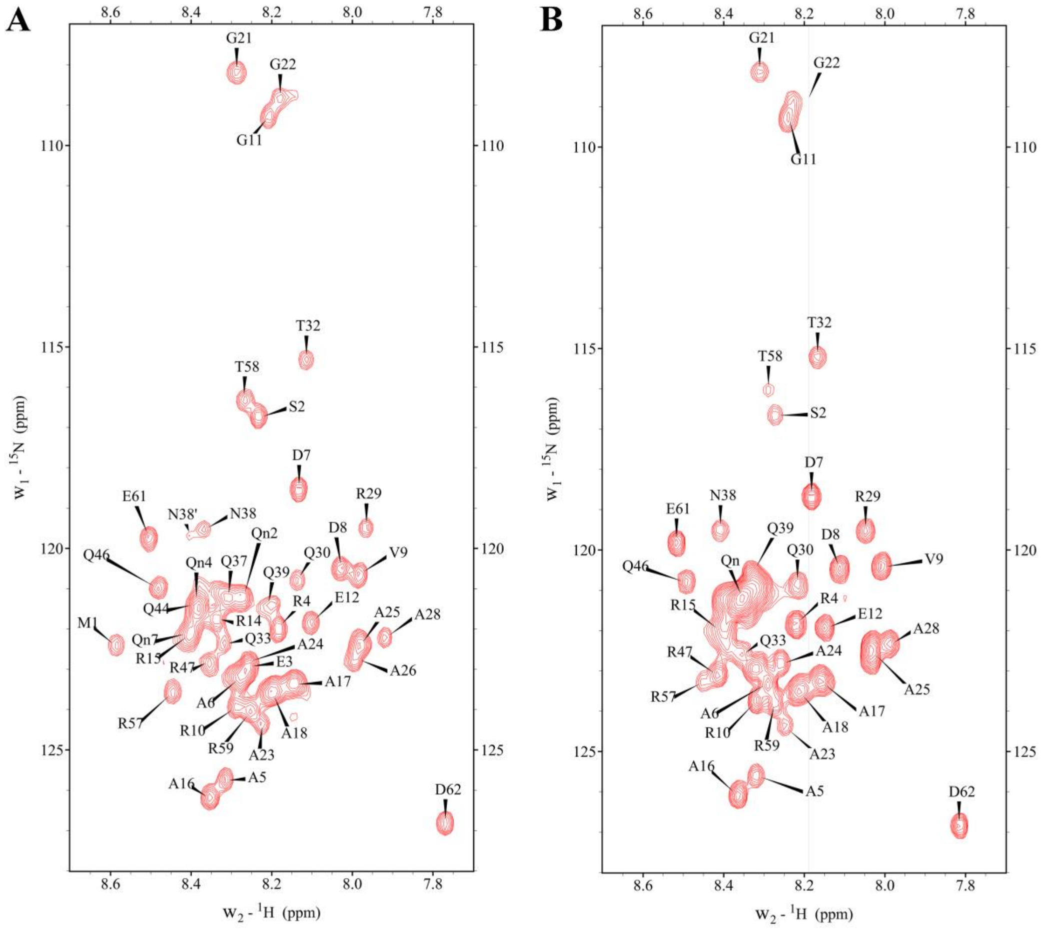

Figure S5. The ^{1}H-^{15}N HSQC spectra of the T3N9 mutants showing the chemical shift assignments. (**A**) Atx7$_{10Q}$-N (T3N9). (**B**) Atx7$_{22Q}$-N (T3N9).

Suppl. Table S1. List of all the constructs used in this study.

Constructs	Vectors	Restriction Enzyme sites	Primers Forward (F) and Reversed (R)
pET-32M-Trx- Atx7$_{10Q}$-N	pET-32M	BamHI / XhoI	F: CGCGGATCCATGTCGGAGCGGGCCGCG;<br>R: CCGCTCGAGTTAGTCCTCCGGCCGTGTGCG
pET-32M-Trx- Atx7$_{22Q}$-N	pET-32M	BamHI / XhoI	F: CGCGGATCCATGTCGGAGCGGGCCGCG;<br>R: CCGCTCGAGTTAGTCCTCCGGCCGTGTGCG
pET-32M-Trx- Atx7$_{33Q}$-N	pET-32M	BamHI / EcoRI	F: CGCGGATCCATGTCGGAGCGGGCCGCG;<br>R: CCGCTCGAGTTAGTCCTCCGGCCGTGTGCG
pET-32M-Trx- Atx7$_{43Q}$-N	pET-32M	BamHI / XhoI	F: CGCGGATCCATGTCGGAGCGGGCCGCG;<br>R: CCGCTCGAGTTAGTCCTCCGGCCGTGTGCG
pET-32M-Trx- Atx7$_{10Q}$-N (T3N9)	pET-32M	BamHI / XhoI	F: GCCCGGCAGCAGACGCAGCAGCAGCAGCAGAATCAGCCGCCGCCT;<br>R: AGGCGGCGGCTGATTCTGCTGCTGCTGCTGCGTCTGCTGCCGGGC
pET-32M-Trx- Atx7$_{22Q}$-N (T3N9)	pET-32M	BamHI / XhoI	F: GCCCGGCAGCAGACGCAGCAGCAGCAGCAGAATCAGCCGCCGCCT;<br>R: AGGCGGCGGCTGATTCTGCTGCTGCTGCTGCGTCTGCTGCCGGGC
pET-32M-Trx- Atx7$_{33Q}$-N (T3N9)	pET-32M	BamHI / XhoI	F: GCCCGGCAGCAGACGCAGCAGCAGCAGCAGAATCAGCCGCCGCCT;<br>R: AGGCGGCGGCTGATTCTGCTGCTGCTGCTGCGTCTGCTGCCGGGC
pET-32M-Trx- Atx7$_{33Q}$ -N (A26G)	pET-32M	BamHI / XhoI	F: GGCGGAGCAGCGGCCGGGGCCGCCCGGCAGCAG;<br>R: CTGCTGCCGGGCGGCCCCGGCCGCTGCTCCGC
pET-32M-Trx- Atx7$_{33Q}$-N (A26P)	pET-32M	BamHI / XhoI	F: GGCGGAGCAGCGGCCCCCGCCGCCCGGCAGCAG;<br>R: CTGCTGCCGGGCGGCGGGGGCCGCTGCTCCGCC
pET-32M-Trx- Atx7$_{33Q}$-N (Δ25-27)	pET-32M	BamHI / XhoI	F: GCGGCGGCGGCGGGCGGAGCAGCCGCCCGGCAGCAGCAGCAGCAG;<br>R: CTGCTGCTGCTGCTGCCGGGCGGCTGCTCCGCCCGCCGCCGCCGC
FLAG-pcDNA3.1-Atx7$_{33Q}$-N172	pcDNA3.1	BamHI / XhoI	F: GCGGGATCCATGTCGGAGCGGGCC;<br>R: CCGCTCGAGATGAGCTATGTCTTC
FLAG-pcDNA3.1-Atx7$_{33Q}$-N172 (A26G)	pcDNA3.1	BamHI / XhoI	F: GGCGGAGCAGCGGCCGGGGCCGCCCGGCAGCAG;<br>R: CTGCTGCCGGGCGGCCCCGGCCGCTGCTCCGC
FLAG-pcDNA3.1-Atx7$_{33Q}$-N172 (A26P)	pcDNA3.1	BamHI / XhoI	F: GGCGGAGCAGCGGCCCCCGCCGCCCGGCAGCAG;<br>R: CTGCTGCCGGGCGGCGGGGGCCGCTGCTCCGCC
FLAG-pcDNA3.1-Atx7$_{33Q}$-N 172 (Δ25-27)	pcDNA3.1	BamHI / XhoI	F: GCGGCGGCGGCGGGCGGAGCAGCCGCCCGGCAGCAGCAGCAGCAG;<br>R: CTGCTGCTGCTGCTGCCGGGCGGCTGCTCCGCCCGCCGCCGCCGC
